# Quality of Life and Cost Study of Rheumatoid Arthritis Therapy With Biological Medicines

**DOI:** 10.3389/fphar.2018.00794

**Published:** 2018-07-18

**Authors:** Vladimira V. Boyadzieva, Nikolay Stoilov, Rumen M. Stoilov, Konstantin Tachkov, Maria Kamusheva, Konstantin Mitov, Guenka I. Petrova

**Affiliations:** ^1^Faculty of Medicine, University Hospital St. Ivan Rilski, Medical University of Sofia, Sofia, Bulgaria; ^2^Faculty of Pharmacy, Medical University of Sofia, Sofia, Bulgaria

**Keywords:** Rheumatoid arthritis, biological therapy, cost-effectiveness, EQ5D, quality of life

## Abstract

Biological medicines are considered as a cornerstone in the therapy of rheumatoid arthritis (RA). They change the course of the disease and improve the quality of life of patients. To this date there has been no study comparing the quality of life of and cost of RA therapy in Bulgaria. This fact is what provoked our interest toward this research. The aim of this study is to analyse the cost and quality of life of patients with RA threated with biological medicines in Bulgaria. This is an observational, real life study of 124 patients treated with biological medicines during 2012–2016 at the University hospital “St. Ivan Riskli” in Sofia, specialized in rheumatology disease therapy. Patients were recruited after their consecutive transfer from non-biological to biological medicines. The yearly pharmacotherapy cost was calculated with tocilizumab (*n* = 30), cetrolizmab (*n* = 16), golimumab (*n* = 22), etanercept (*n* = 20), adalimumab (*n* = 20), rituximab (*n* = 16). Three measurements of the quality of life (QoL) were performed with EQ5D—at the beginning of the therapy, after 6 months and after 1 year of therapy. Both section of EQ5D were used—VAS and EQ5D questionnaire. Cost—effectiveness was calculated for unit of improvement in EQ5D score for a one year period and decision model was built with TreeAgePro software. The observed cost of therapy varied between 12 thousand Euros for tocilizumab to 6 thousand Euros for rituximab. All biological medicines let to substantial increase in the quality of life of the patients. Patients on tocilizumab increased their QoL from 0.43 to 0.63 after 1 year; on cetrolizumab from 0.32 to 0.56; on golimumab from 0.41 to 0.67; on etanercept from 0.45 to 0.62; on adalimumab from 0.43 to 0.57; on rhituximab from 0.46 to 0.66. The cost-effectiveness estimates of different biological therapies also varied between 66 to 30 thousand Euros for unit of improvement in the EQ5D during one the course of the year. Therapy with biological medicines improves statistically significant the quality of life of patients, measured through VAS and EQ5D scales. Despite the improvement in the quality of life all biological medicines appears not to be note cost-effective due to their high incremental cost-effectiveness ration (ICER). Rituximab's incremental ratio has (ICER) falls closer to the three times gross domestic product per capita threshold and should be considered as preferred alternatives for RA therapy. In general we can conclude that the treatment of rheumatoid arthritis with biologicals improves quality of life significantly. Only rituximab was cost-effective.

## Introduction

Rheumatoid arthritis (RA) is the most common, chronic, autoimmune disease with world prevalence of 0.5–1% of the population (Woolf and Pfleger, 2003). In central and Eastern Europe prevalence estimates are: 0.37% for Hungary among people aged 14–65, for the Czech Republic 0.61%, Estonia 0.46%, and out of them 50–55% of the people are in productive ages (Kiss et al., 2005). In Romania the prevalence is 0.2% among males and 0.5% among females, in Russia around 0.68% (Orlewska et al., 2011). For Bulgaria in 2008 registered patients with RA numbered 29 711 (0.4%) (Kobelt and Kasteng, 2009).

RA affects mortality and quality of life of patients (Blumenauer et al., 2003; Haroon et al., 2007). The average life expectancy is 4 years lower for males with RA and 10 years for females than their respective averages. In patients with severely affected mobility, acute form of RA, visceral manifestation, and concomitant diseases the mortality rate is higher (Kvien, 2004).

Biological medicines are considered as a cornerstone therapy of rheumatoid arthritis (RA) for patients that do not respond to methotrexate or other disease modifying agents (Felson et al., 1993, 1995). Biologicals change the course of the disease, improve the quality of life of the patients, and decrease mortality (Felson et al., 2011).

The term biologic medicines include adalimumab, certolizumab pegol, etanercept, golimumab, infliximab which belong to TNF alfa inhibitors class, as well as medicines acting through different mechanism such as abatacept, anakinra, rituximab, and tocilizumab. All are recognized as an effective treatment for RA, but they are usually recommended only for patients with insufficient response or intolerance to synthetic disease modifying agents, due to price concerns by authorities (Nam et al., 2010, 2014; Aaltonen et al., 2012; Smolen et al., 2014).

There are numerous studies of the cost-effectiveness of biologic therapy but real life studies are limited (Cooper, 2000; Joensuu et al., 2015). There is also no study comparing the quality of life of and cost of RA therapy in Bulgaria in real life settings. The gap in knowledge provoked our interest toward this research. Therefore the aim of this study is to analyse the cost and quality of life of patients with RA threated with biological medicines in Bulgaria.

## Materials and methods

This is an observational, real-life study of 124 patients treated with biological medicines during 2012–2016 at the University hospital “St. Ivan Riskli” in Sofia, specialized in rheumatology disease therapy.

Patients were consecutively recruited after their transfer from non-biological to biological therapy and followed for 1 year.

Inclusion criteria were: age above 18 years; willingness to participate after informed consent; confirmed diagnosis of RA according ACR/EULAR (2010) (Aletaha et al., 2010); treatment naïve on biological therapy; previous treatment with methotrexate and non-steroidal anti-inflammatory drugs or methotrexate and other disease modifying therapy; adherence to therapy in the previous 6 months and during the whole period of observation. Exclusion criteria were infectious diseases (HIV, tuberculosis); cardiac insufficiency (NYHA III and IV grade); malignant hypertension; psychiatric diseases; any neoplasms or proliferative lymph diseases within the previous 5 years (Dolgin et al., 1994); alcohol or narcotic abuse; deficiencies in recognition abilities. On total 110 female and 14 male were recruited.

The quality of life was assessed through the EQ5D questionnaire (Devlin, 2017). Both section of EQ5D were used – VAS and EQ5D questionnaire. The measurements of the quality of life (QoL) were performed at the beginning of the therapy, after 6 months, and after 1 year. Then the changes in the QoL were evaluated statistically.

Physicians choose the biological medicines according to their personal opinion based on corresponding clinical status of the patients and available medicines in the reimbursement list. On the basis of physicians' choice, the patients were separated in groups of prescribed biologic products for the purposes of the analysis. The following groups were formed: tocilizumab (*n* = 30), cetrolizmab (*n* = 16), golimumab (*n* = 22), etanercept (*n* = 20), adalimumab (*n* = 20), rituximab (*n* = 16). The changes in QoL were compared among groups of patients for both scales VAS and EQ5D questionnaire.

Ethical committee of the University hospital “St. Ivan Riskli” in Sofia approved the study.

The yearly pharmacotherapy cost for the corresponding medicine was calculated by multiplying the official price per defined daily dose gathered from National council on prices and reimbursement by 365 days^1^. The prices were collected at the end of 2016 and expressed in national currency (BGN). The exchange rate is 1 Euro = 1.958 BGN.

To evaluate the cost effectiveness of the products we built a decision tree model with TreeAgePro software comparing the yearly pharmacotherapy cost with the changes in EQ5D scores after 1 year of therapy. Probability of prescribing a particular INN was derived from our sample.

## Results

### Changes in the quality of life (QoL)

Statistically significant QoL increases were observed for both EQ5D scales (Table 1). Patients' self-assessment of their health state using the visual analogs scale (VAS) grew from 44 to 76 point out of 100 maximum possible. Similar increases were observed in the combined EQ5D evaluation—from 0.42 to 0.624 points.

**Table 1:** Changes in the QoL during the observation for all patients.

**Scale**	**Beginning of therapy**	**After 6 months**	**After 1 year**	***P***
Visual analog scale(VAS) - Mean Value (95% CI)	44.056 (41.220–46.893)	63.242 (60.306–66.178)	76.379 (73.739–79.019)	<0.00001
EQ5D- Mean Value (95% CI)	0.420 (0.386 to 0.455)	0.5896 (0.216 to 0.643)	0.624 (0.595 to 0.653)	<0.0001

Overall, self-evaluation by VAS per INN increased significantly but none of the measurements revealed statistically significant differences when comparing different INNs with each other. Therefore, none of the changes in the QoL per INN are significant (Table 2).

**Table 2:** VAS scores for different INNs.

**INN**	**VAS median value beginning of therapy**	**VAS median value after 6 months therapy**	**VAS median value after 1 year therapy**
Adalimumab	40.00	66.50	80.00
cetrolizumab-pegol	43.50	57.00	77.50
Etanercept	43.50	65.00	75.00
Golimumab	42.50	69.00	80.50
Rituximab	50.00	69.50	82.50
Tocilizumab	41.50	69.50	85.00
Kruskal-Wallis test – p	0.782038	0.670970	0.670249

Similar were the results for EQ5D domains where there was no difference among the scores between the medicines (Table 3). For adalimumab and cetrolizumab, no changes in the EQ5D score were observed after second and third measurement.

**Table 3:** EQ5D scores for different INNs.

**INN**	**EQ5D median value beginning of therapy**	**EQ5D median valueafter 6 months therapy**	**EQ5D median value after 1 year therapy**
Adalimumab	0.480	0.560	0.560
cetrolizumab-pegol	0.241	0.570	0.570
Etanercept	0.480	0.570	0.620
Golimumab	0.480	0.586	0.630
Rituximab	0.480	0.598	0.660
Tocilizumab	0.480	0.570	0.670
Kruskal-Wallis test - p	0.526611	0.715242	0.318079

Male patients reported lower QoL than female patients (Table 4) although for both groups the VAS and EQ5D scale showed a statistically significant increase in their scores.

**Table 4:** Differences in QoL between male and female group.

**Scale**	**Beginning of therapy**	**After 6 months**	**After 1 year**	***P***
**FEMALE GROUP**				
Visual analog scale(VAS) - median (min to max)	45.000 (10.00–80.00)	68.000 (5.00–100.000)	80.000 (30.000–100.000)	<0.00001
EQ5D- Mean Value (95% CI)	0.425 (0.389–0.461)	0.589 (0.559–0.619	0.622 (0.591–0.652)	<0.0001
**MALE GROUP**				
Visual analog scale(VAS) - median (min to max)	33.50 (10.000–75.000)	57.000 (30.000–85.000)	79.000 (46.000–100.000)	<0.00001
EQ5D- Mean Value (95% CI)	0.387 (0.260 to 0.514)	0.5935 (0.48–0.650)	0.640 (0.530–0.750)	<0.0001

### Cost-effectiveness analysis

**Figure 1:**
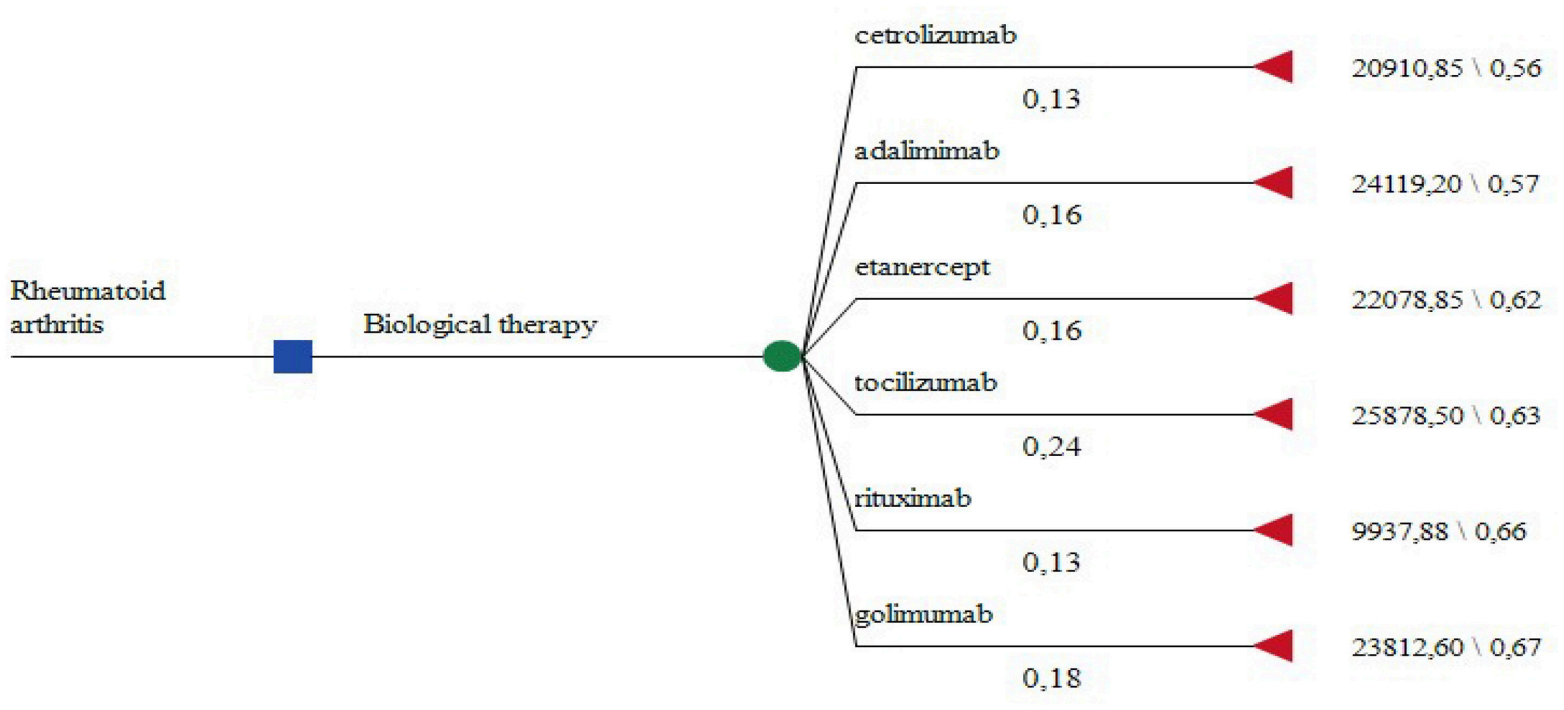
The decision tree model.

Due to the lack of statistical significance in the EQ5D scores between the prescribed INNs we consider the therapeutic results achieved as identical. Therefore we constructed a decision tree model where the choice of biologic therapy depends only on the subjective physician opinion and he/she could choose any one of the available on the local market alternatives (Figure 1). The probability of prescribing a particular INN is derived from our patient sample.

The yearly cost of therapy with a particular biological is calculated for every INN and the lowest is with rituximab (Table 5). EQ5D scores at the end of therapy are measures of effectiveness.

**Table 5:** Results of the cost-effectiveness calculation.

	**Cost**	**Incremental cost**	**Effectiveness**	**Incremental effectiveness**	CER^*^/ICER^**^
Cetrolizumab	20910,85	20910,85	0.56	0.56	3,734,080
Adalimumab	24119,20	3208,35	0.57	0.01	32,083,500
Etanercept	22078,85	−2040,35	0.62	0.05	−4,080,700
Tocilizimab	25878,50	3799,65	0.63	0.01	37,996,500
Rituximab	9937,88	−15940,62	0.66	0.03	−53,135,400
Golimumab	23812,60	13874,72	0.67	0.01	138,747,200
	**Cost**	**Incremental cost**	**Effectiveness**	**Incremental effectiveness**	**CER/ICER**
Adalimumab	24,119,20	241,192	0.57	0.57	4,231,439
Etanercept	2,207,885	−204,035	0.62	0.05	−4,080,700
Tocilizimab	2,587,850	379,965	0.63	0.01	37,996,500
Rituximab	993,788	−1,594,062	0.66	0.03	−53,135,400
	**Cost**	**Incremental cost**	**Effectiveness**	**Incremental effectiveness**	**CER/ICER**
Etanercept	2,207,885	2,207,885	0.62	0.62	3561,105
Rituximab	993,788	−1,214,097	0.66	0.04	−30,352,425

* CER (cost-effectiveness ration = cost of INN/effectiveness of INN). Used only for the first alternative.

** *ICER (Incremental cost—effectiveness ration = (Cost_a_−Cost_b_)/(Effectiveness_a_−Effectiveness_b_). For every INN is calculated the difference between the cost and effectiveness with the previous one INN in the table*.

Applying the rules for incremental cost effectiveness ratio calculation (ICER) rituximab appears to be the most cost-effective alternative, followed by etanercept, tocilizumab, and adalimumab. Cetrolizumab is less effective and golimumab is not cost-effective (Table 5) despite its higher effectiveness.

**Figure 2:**
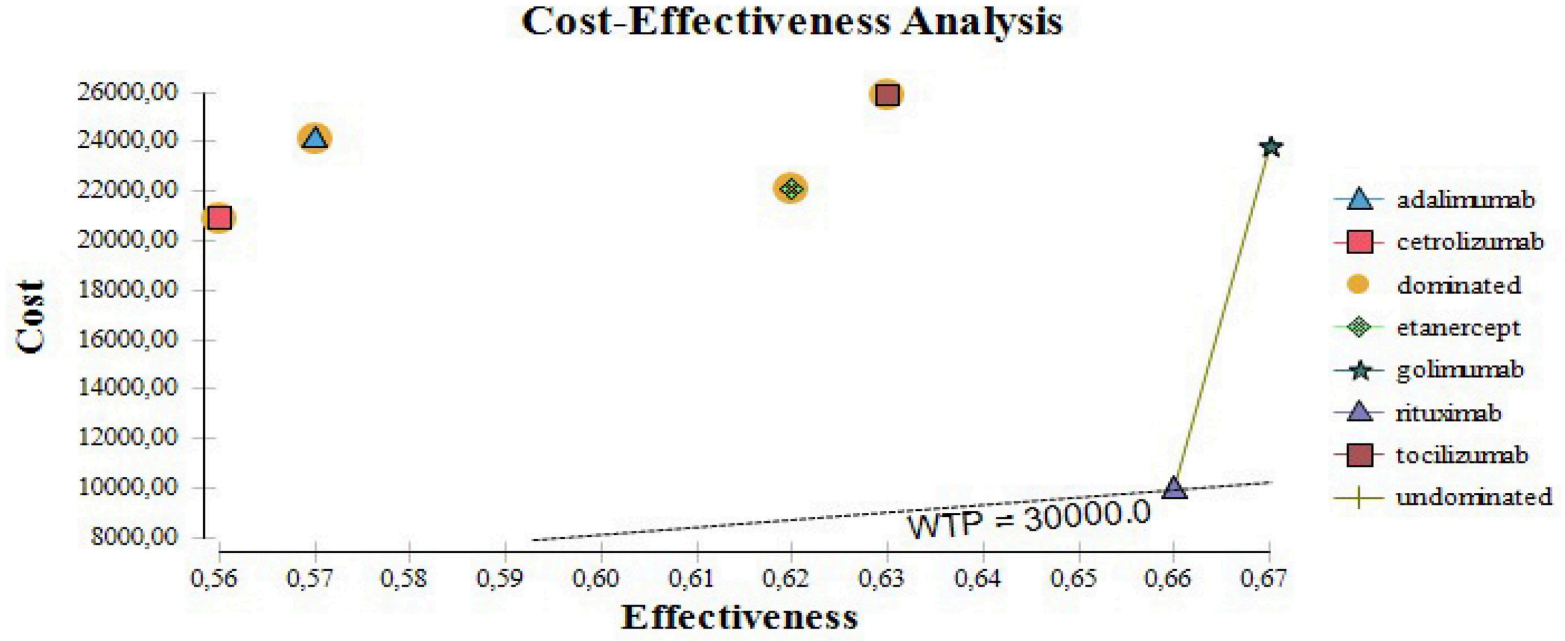
Cost effectiveness plate for biological medicines.

Cetrolizumab, adalimumab, golimumab, and tocilizumab are all dominated by rituximab. The cost-effectiveness of biological therapy varies from 30 to 66 thousand BGN for unit of improvement in the EQ5D after one year of therapy. If the willingness to pay threshold is 30 000 only for rituximab the ICER is below that threshold (Figure 2).

## Discussion

Improvement in the quality of life of individual patients with RA is one of the main goals of therapy because of its serious rate of deterioration (Jørgensen et al., 2017). Our study confirms that the QoL measured with EQ5D improves quickly in the beginning of therapy and subsequently slows down toward the end of one year of therapy. Despite this, overall improvement is statistically significant. Similar results were reported in other articles although in our study we observed slightly higher EQ5D scores (Pollard et al., 2005; Pickard et al., 2007; Aaltonen et al., 2012; Kim et al., 2015).

EQ-5D was chosen as utility outcome because of its simplicity, wide-spread use and well-established scores. To the best of our knowledge this is the first national study of the QoL of patients with RA that uses the EQ5D.

Our study also shows that males reported lower quality of life than females. It could be due to different perception between genders toward pain and suffering (Wijnhoven et al., 2007). A reason for lower values could also be the small male sample. Many studies report that RA affects mostly women, which was confirmed in our study results for Bulgaria (Woolf and Pfleger, 2003; Kiss et al., 2005).

We found no differences in the changes of the QoL with different INNs of biological medicines after one year therapy. Similar results were reported in a systematic review and meta-analysis, published in 2011 (Malottki et al., 2011). This could be because real-life settings influence the results when measuring, which could account for the disparity when compared to results published from randomized clinical trials (Gülfe et al., 2010). The lack of statistically significant differences allows building a decision tree model that reflects real life therapy, based on the probability for an individual physician's choice. Other studies have also selected rituximab as a cost-effective alternative (Pollard et al., 2005; Joensuu et al., 2015). In general treatment of rheumatoid arthritis with biologicals improves quality of life significantly. Only rituximab was cost-effective. These results could be used by health authorities to optimize RA therapy and better control prescription of biologics.

The limitations are the overall low number of participants, especially the number of male patients, as well as the number in each different biological group. It is also a single center study. The strengths are the quality of the data.

Further analysis should be done when new biosimilars appear on the market and medicines prices change.

## Conclusions

Therapy with biological medicines improves statistically significant the quality of life of patients, measured through VAS and EQ5D scales. Despite the improvement in the quality of life all biological medicines appear not to be cost-effective due to their high incremental cost-effectiveness ratio (ICER). Rituximab's incremental ratio (ICER) falls closer to the three times gross domestic product per capita threshold and should be considered as a preferred alternative for RA therapy. In general we can conclude that the treatment of rheumatoid arthritis with biologicals improves quality of life significantly. Only rituximab was cost-effective.

## Ethics statement

Ethic committee of the University hospital St. Ivan Rislki.

## Author contributions

VB, NS, and RS design the study and recruited patients. KT and MK evaluate cost-effectiveness. KM did statistical analysis. GP wrote article and design the manuscript. All authors approved the manuscript.

### Conflict of interest statement

The authors declare that the research was conducted in the absence of any commercial or financial relationships that could be construed as a potential conflict of interest.

## Footnotes

^1^National council on prices and reimbursement. Prices registry. Available online at: www.ncpr.bg
